# Artificial autotetraploidy modulates flowering regulation in industrial hemp through DNA methylation and transcriptomic reprogramming

**DOI:** 10.1186/s43897-026-00245-8

**Published:** 2026-08-06

**Authors:** Chen Kuang, Ying Xu, Boyang Liu, Chaohua Cheng, Canhui Deng, Jianguang Su, Xiaoyu Zhang, Zhigang Dai, Zemao Yang, Jiquan Chen, Ying Xu, Qing Tang

**Affiliations:** 1 Chinese Academy of Agricultural Sciences Center for Southern Economics Corps, Chinese Academy of Agricultural Sciences Institute of Bast Fiber Crops, Changsha, Hunan 410205 People’s Republic of China; 2 National Breeding Center for Bast Fiber Crops, Changsha, Hunan 410205 People’s Republic of China; 3 Yuelushan Laboratory, Changsha, Hunan 410128 People’s Republic of China

Whole-genome duplication (WGD) is a major evolutionary force shaping plant diversity and agronomic traits, often leading to pronounced changes in morphology, development, and environmental adaptability (Chen 2007; Leslie & Mander 2025; Wolfe et al. 2023). In many crops, polyploidization has been associated with increased organ size, enhanced biomass accumulation, and altered reproductive timing (Chen 2007; Qiao et al. 2019; Tank et al. 2015). However, the molecular mechanisms by which autopolyploidization reshapes developmental programs, particularly flowering regulation, remain incompletely understood in industrial hemp (*Cannabis sativa* L.). Here, by integrating phenotypic observations with genome-wide DNA methylation, transcriptome, and small RNA analyses, we demonstrate that auto-tetraploidization in cannabis establishes a multilayered regulatory network centered on circadian rhythm genes and flowering integrators, ultimately leading to delayed flowering and increased vegetative biomass (Ma et al. 2025; Zhang et al. 2025).

Photoperiod regulation reveals morphological advantages and delayed flowering response with enhanced biomass yield in tetraploid cannabis (Fig. 1A–F; Fig. S1 and Table S1). Cytological analysis and flow cytometry confirmed that the induced plants were true autotetraploid with 2n = 40 chromosomes (Fig. S1). Compared with diploid plants, tetraploid cannabis exhibited pronounced morphological enlargement, including significantly increased leaf dimensions and inflorescence size (Fig. 1B–C). Under short-day conditions, tetraploid plants showed sustained stem elongation, whereas diploid plants rapidly ceased vegetative growth (Fig. S1). Floral initiation and subsequent flowering stages were consistently delayed in tetraploids, indicating altered photoperiodic responsiveness (Fig. S1 and S2). These developmental shifts were accompanied by substantial increases in inflorescence and whole-plant biomass, highlighting the yield advantages conferred by tetraploidization. Paraffin section analysis revealed conserved floral organogenesis in diploid and tetraploid industrial hemp, but with distinct developmental timing differences (Fig. S2). Diploid plants initiated floral primordia and ovule development earlier, whereas tetraploid plants showed delayed early differentiation but developed larger and more densely packed floral tissues at later stages (Fig. 1D–E and Fig. S2). These findings suggest that polyploidization suppresses early floral bud development while enhancing late-stage reproductive growth.

**Fig. 1:**
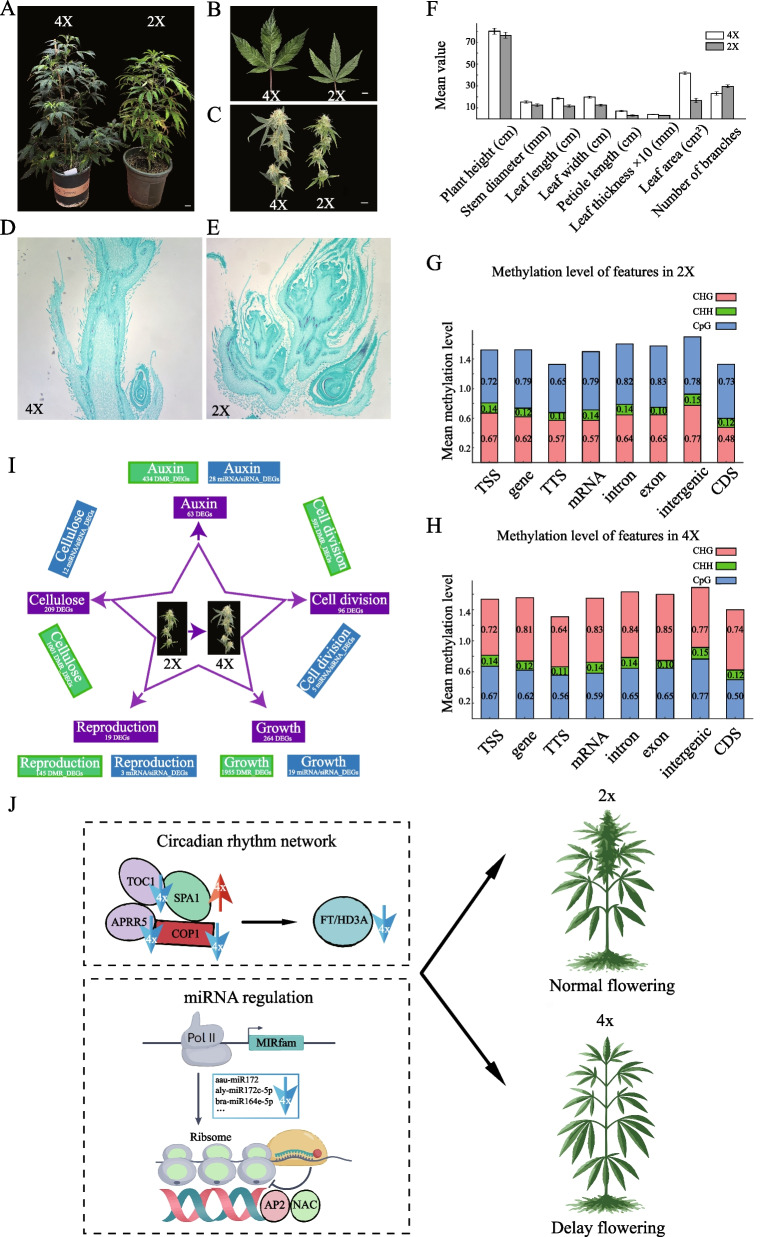
Phenotypic Traits and Molecular Impact in Tetraploid and Diploid Variants of Industrial Hemp. **A** Morphological characteristics of vegetative growth in tetraploid (left) and diploid (right) industrial hemp plants, with a scale bar of 5 cm; **B** Leaf morphology of hemp, with a scale bar of 1 cm; **C** Morphology of mature hemp inflorescence, with a scale bar of 1 cm; **D**–**E** Autotetraploid and diploid industrial hemp flower bud paraffin sections at initial flowering period; **F** Statistical analysis of hemp phenotypes traits; **G**–**H** Relative proportions of CHG, CHH and CpG mode for TSS, gene, TTS, mRNA, intron, exon, intergenic, and CDS regions; **I** Numbers of differentially expressed transcripts potentially regulated by DMRs and small RNAs, categorized into five major plant growth processes: auxin response, cell division, cellulose metabolic/biosynthetic processes, growth, and reproduction. **J** Schematic diagram of our hypothetical model integrating circadian rhythm network and miRNA regulation in Flowering time of tetraploid (4X) and diploid (2X) hemp. The blue arrow denotes downregulation of the respective gene or miRNA, while red indicates upregulation. MiRfam represents a miRNA gene family. A blocked line ('T') indicates inhibition

Identification of differentially methylated regions contributing to phenotypic variations following homologous tetraploidization (Fig. 1G–H). Whole-genome bisulfite sequencing showed that CHH methylation represented the major methylation context in both diploid and tetraploid cannabis, with no substantial changes in overall context proportions (Fig. S3). However, tetraploid plants displayed elevated CpG and CHG methylation across gene-associated regions, accompanied by a pronounced reduction of CHH methylation, particularly around transcription start sites and within gene bodies (Fig. 1G–H and Fig. S3). This context-specific epigenetic remodeling suggests that homologous tetraploidization selectively reshapes regulatory-region methylation to facilitate transcriptional reprogramming underlying polyploid phenotypic variation. Epigenetic profiling of differentially methylated regions (DMRs) following whole-genome duplication in *Cannabis sativa* revealed significant changes in methylation across CG, CHG, and CHH contexts, with tetraploid plants exhibiting increased methylation in CpG and CHG regions and decreased CHH methylation in regulatory regions (Fig. S4). GO enrichment analysis indicated that these methylation changes are enriched in pathways related to developmental processes such as embryonic development, oxylipin biosynthesis, and reproductive regulation (Fig. 1I and Supplementary Data S1-S9). These results underscore the role of methylation remodeling in modulating key genes responsible for phenotypic variation in tetraploid hemp.

Transcriptomic variations of Small RNAs and differentially expressed genes following autotetraploid in industrial hemp (Fig. S5). Transcriptome analysis revealed that 89 differentially expressed miRNAs and 21-nt/24-nt siRNAs target genes involved in key pathways, including auxin response, cell division, and cellulose metabolism, in tetraploid *Cannabis sativa* (Fig. S5). Overlap analysis between differentially methylated regions (DMRs) and differentially expressed genes (DEGs) highlighted significant associations, with 410 genes shared between DMRs and DEGs, and 25 genes co-regulated by miRNAs/siRNAs (Fig. S5 and Supplementary Data S1-S9). These results underscore the role of small RNAs and epigenetic modifications in regulating key genes that drive phenotypic variation following polyploidization in hemp.

Polyploidization induced pronounced morphological and developmental changes in industrial hemp, including delayed flowering, expanded leaf area, increased thickness, and enhanced biomass accumulation (Fig. 1A–F). These phenotypic shifts prompted an integrated analysis of epigenetic and transcriptional regulation following whole-genome duplication. Several key circadian and flowering regulators, including *HD3A(FT)*, *TOC1*, *APRR5*, *SPA1*, and *COP1*, were concurrently differentially expressed and differentially methylated in tetraploid plants (Fig. S6). Repression of *HD3A* was associated with increased promoter methylation, consistent with its central role in photoperiodic flowering control. Similarly, reduced expression of *TOC1* and *APRR5* was partially restored by DNA demethylation, indicating direct epigenetic regulation of the circadian network (Sato et al. 2002). Light-signaling components *SPA1* and *COP1* further contributed to suppression of *FT*-dependent flowering pathways. Moreover, tetraploid cannabis showed significant variations in small RNA-mediated regulation, with a shift in miRNA expression profiles targeting key transcription factors like AP2, NAC, and others (Fig. S5C and Fig. 1J). The downregulation of miR172 and miR164 allowed for increased levels of AP2 and NAC transcription factors, leading to further repression of flowering. Additionally, DNA methylation and small RNA regulation were found to work synergistically, contributing to a stable delay in flowering. These multilayered regulatory changes in tetraploid cannabis reflect a conserved polyploid-associated mechanism observed across plant species. This integrated regulatory network provides valuable insights into the mechanisms underlying the delayed flowering phenotype in tetraploid cannabis, offering new pathways for breeding strategies and crop improvement. These results are consistent with findings in other polyploid species, suggesting that similar molecular principles govern polyploid-induced developmental changes across plants (Yan et al. 2019; Yu et al. 2018). The graphical model further illustrates these interconnections between epigenetic modifications, transcriptional regulation, and phenotypic outcomes in tetraploid cannabis (Fig. 1J). Specifically, genome duplication leads to coordinated repression of core circadian components (TOC1, APRR5, COP1, and FT/HD3A) through DNA methylation–associated transcriptional downregulation, thereby weakening photoperiodic flowering signals. In parallel, reduced accumulation of flowering-related miRNAs (notably miR172 and miR164) alleviates post-transcriptional repression of AP2 and NAC transcription factors, reinforcing suppression of floral transition. Together, these epigenetic and miRNA-mediated regulatory layers converge on the circadian–flowering network, ultimately shifting developmental output from normal flowering in diploids to delayed flowering in autotetraploid plants.

Finally, autotetraploid cannabis possesses considerable potential for interspecific hybridization with closely related species, thereby creating novel germplasm. This extensive genetic recombination capability effectively broadens the genetic resource base, meeting diverse cultivation and market demands. Consequently, autotetraploid breeding approaches promise significant contributions to cannabis germplasm innovation and industry advancement.

## Supplementary Information

Supplementary Material 1.

Supplementary Material 2.

Supplementary Material 3.

Supplementary Material 4.

Supplementary Material 5.

Supplementary Material 6.

Supplementary Material 7.

Supplementary Material 8.

Supplementary Material 9.

Supplementary Material 10.

Supplementary Material 11.

Supplementary Material 12.

Supplementary Material 13.

Supplementary Material 14.

Supplementary Material 15.

Supplementary Material 16.

## Data Availability

Sequencing data are available under NGDC BioProject PRJCA039240. Additional results are provided in Supplemental Information.
