# Supplementary material for: Artificial autotetraploidy modulates flowering regulation in industrial hemp through DNA methylation and transcriptomic reprogramming
Source: Mol Hortic. 2026 Aug 6;6:53. doi: 10.1186/s43897-026-00245-8 (PMC13445816; doi:10.1186/s43897-026-00245-8)
Supplement: Supplementary file 1 [file 43897_2026_245_MOESM1_ESM.pdf]

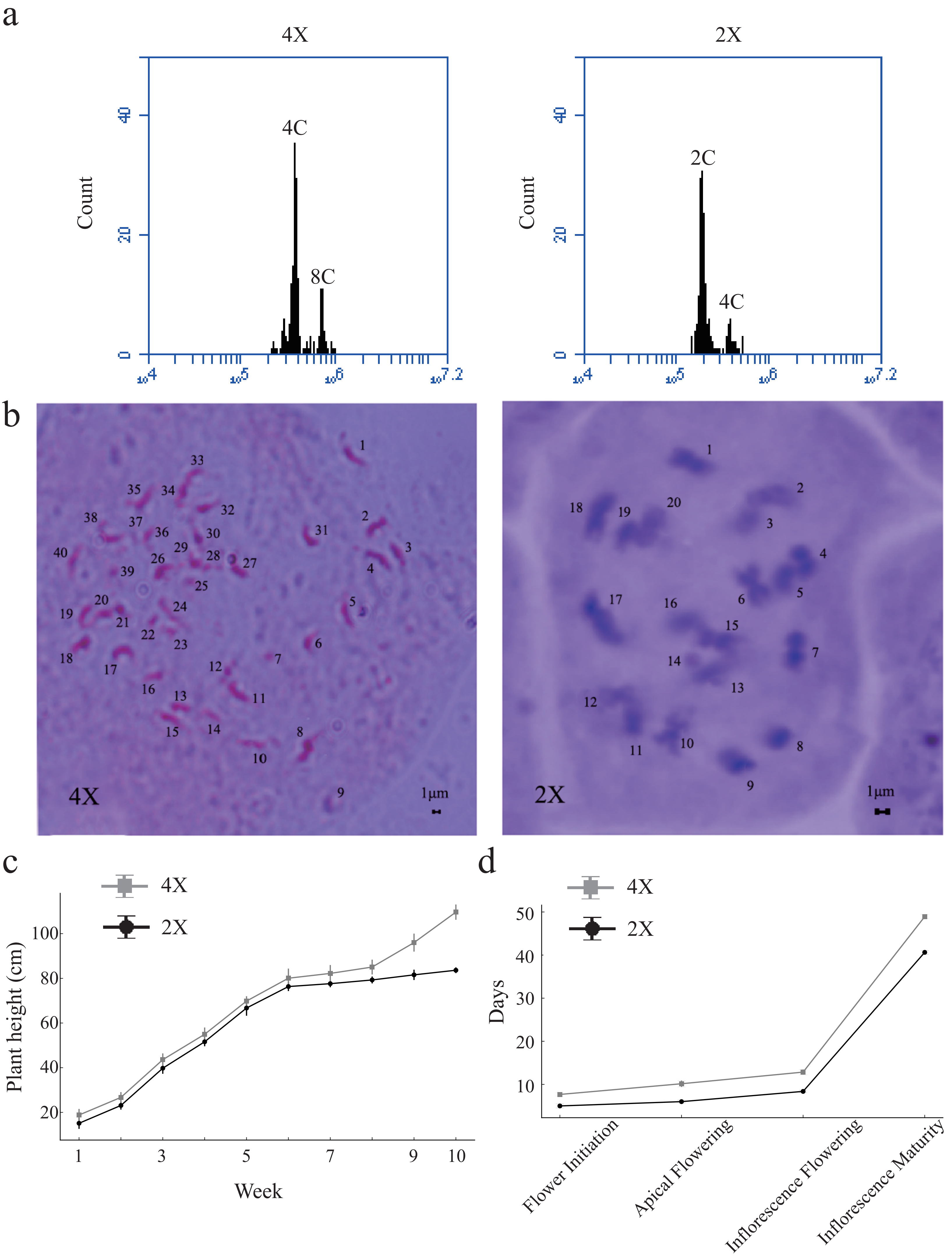

a
4X
2X
Count
40
20
0
4C
8C
2C
4C
10^4
10^5
10^6
10^7.2
b
4X
2X
1μm
1μm
c
4X
2X
Plant height (cm)
Week
d
4X
2X
Days
Flower Initiation
Apical Flowering
Inflorescence Flowering
Inflorescence Maturity
