# Supplementary material for: Artificial autotetraploidy modulates flowering regulation in industrial hemp through DNA methylation and transcriptomic reprogramming
Source: Mol Hortic. 2026 Aug 6;6:53. doi: 10.1186/s43897-026-00245-8 (PMC13445816; doi:10.1186/s43897-026-00245-8)
Supplement: Supplementary file 2 [file 43897_2026_245_MOESM2_ESM.pdf]

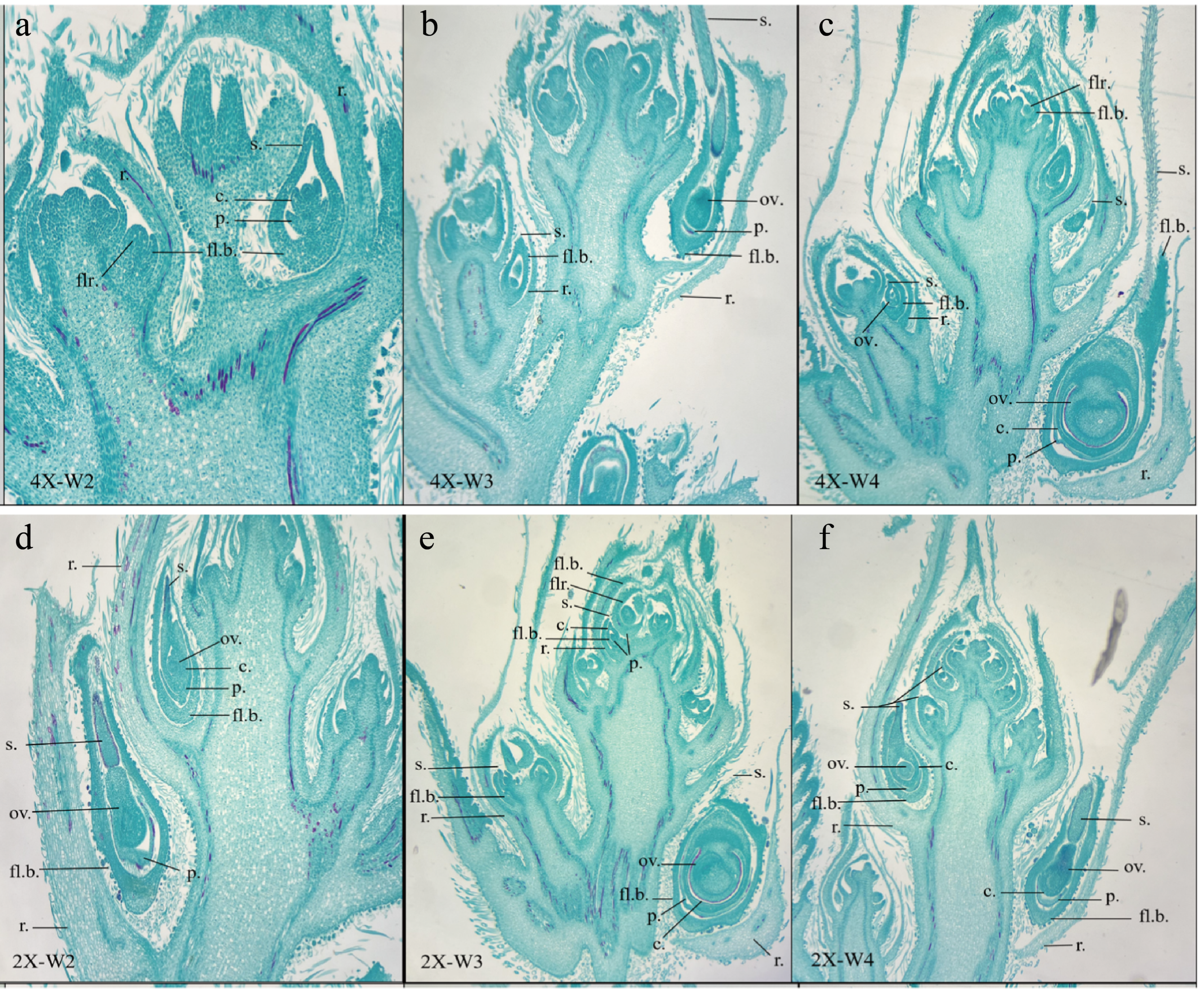
a
r.
s.
r.
c.
p.
fl.b.
flr.
4X-W2
b
s.
ov.
p.
s.
fl.b.
fl.b.
r.
r.
4X-W3
c
flr.
fl.b.
s.
s.
fl.b.
s.
fl.b.
r.
ov.
ov.
c.
p.
r.
4X-W4
d
r.
s.
ov.
c.
p.
fl.b.
s.
ov.
fl.b.
p.
r.
2X-W2
e
fl.b.
flr.
s.
c.
fl.b.
r.
p.
s.
s.
fl.b.
r.
ov.
fl.b.
p.
c.
r.
2X-W3
f
s.
ov.
c.
p.
fl.b.
r.
s.
ov.
c.
p.
fl.b.
r.
2X-W4
