# Supplementary material for: Artificial autotetraploidy modulates flowering regulation in industrial hemp through DNA methylation and transcriptomic reprogramming
Source: Mol Hortic. 2026 Aug 6;6:53. doi: 10.1186/s43897-026-00245-8 (PMC13445816; doi:10.1186/s43897-026-00245-8)
Supplement: Supplementary file 3 [file 43897_2026_245_MOESM3_ESM.pdf]

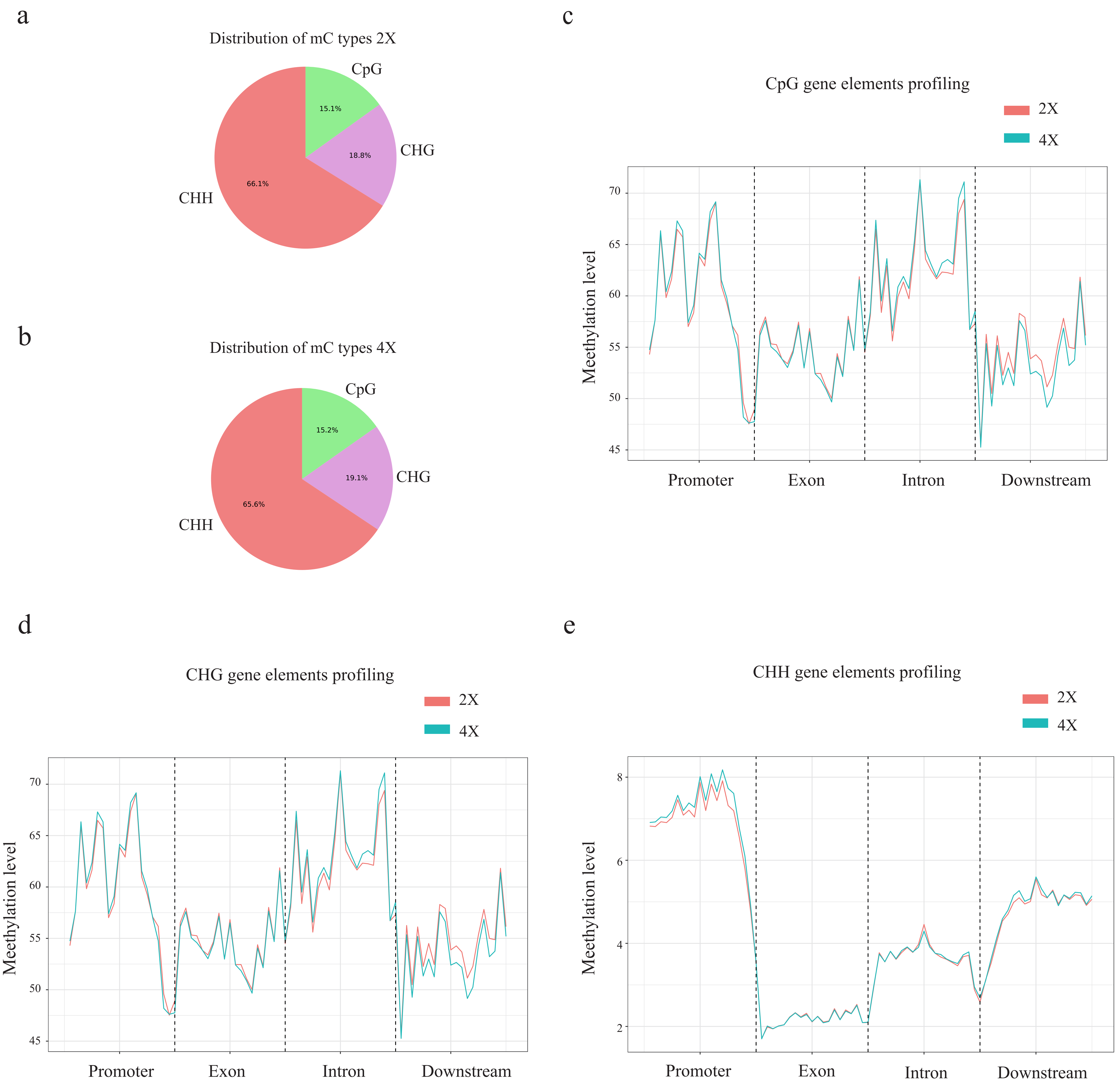
a
Distribution of mC types 2X
CpG
15.1%
CHG
18.8%
CHH
66.1%
b
Distribution of mC types 4X
CpG
15.2%
CHG
19.1%
CHH
65.6%
c
CpG gene elements profiling
2X
4X
Meethylation level
70
65
60
55
50
45
Promoter
Exon
Intron
Downstream
d
CHG gene elements profiling
2X
4X
Meethylation level
70
65
60
55
50
45
Promoter
Exon
Intron
Downstream
e
CHH gene elements profiling
2X
4X
Meethylation level
8
6
4
2
Promoter
Exon
Intron
Downstream
