# Supplementary material for: Artificial autotetraploidy modulates flowering regulation in industrial hemp through DNA methylation and transcriptomic reprogramming
Source: Mol Hortic. 2026 Aug 6;6:53. doi: 10.1186/s43897-026-00245-8 (PMC13445816; doi:10.1186/s43897-026-00245-8)
Supplement: Supplementary file 4 [file 43897_2026_245_MOESM4_ESM.pdf]

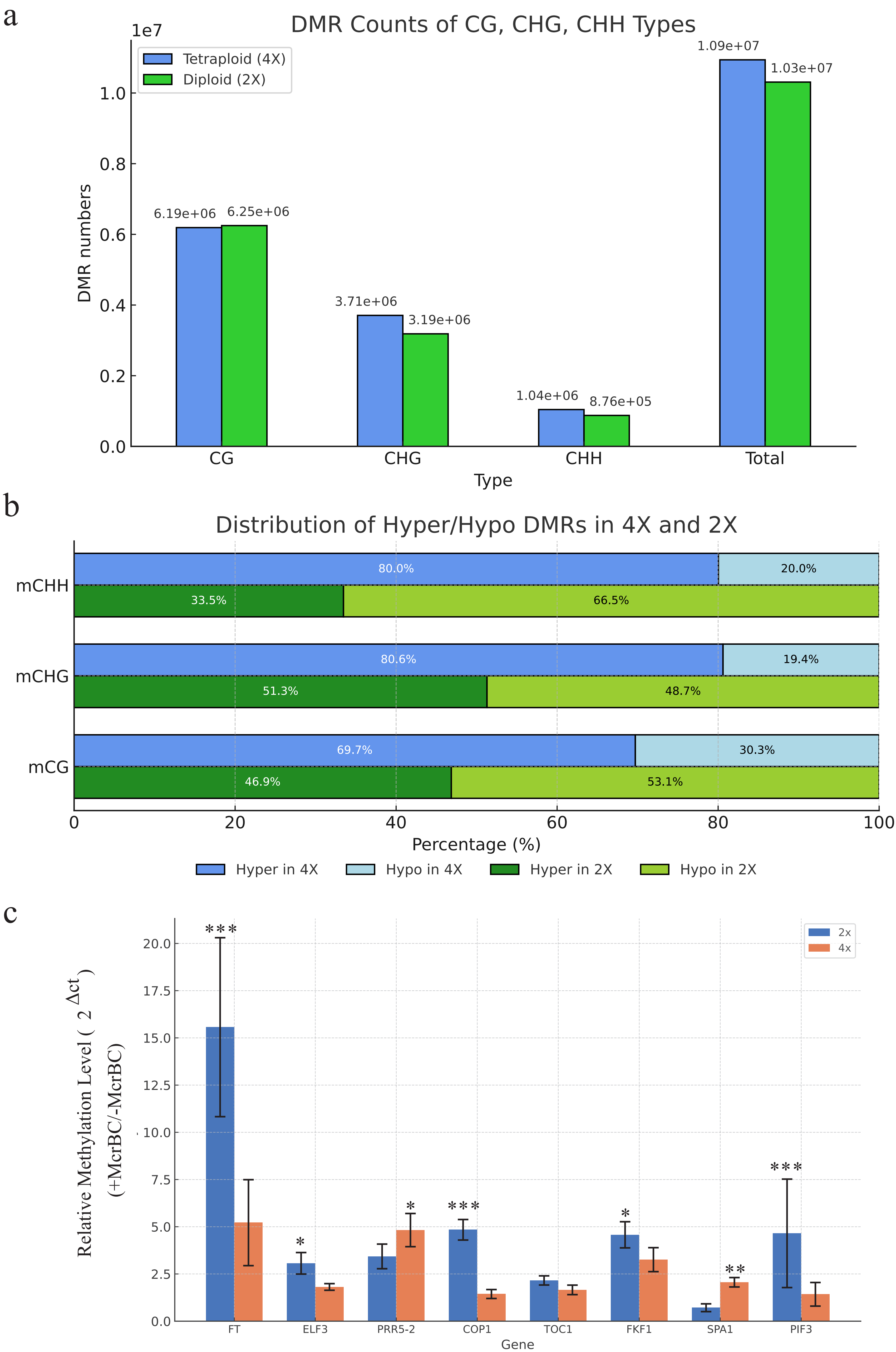
a
DMR Counts of CG, CHG, CHH Types
1e7
Tetraploid (4X)
Diploid (2X)
DMR numbers
0.0
0.2
0.4
0.6
0.8
1.0
6.19e+06
6.25e+06
3.71e+06
3.19e+06
1.04e+06
8.76e+05
1.09e+07
1.03e+07
CG
CHG
CHH
Total
Type
b
Distribution of Hyper/Hypo DMRs in 4X and 2X
mCHH
80.0%
20.0%
33.5%
66.5%
mCHG
80.6%
19.4%
51.3%
48.7%
mCG
69.7%
30.3%
46.9%
53.1%
0
20
40
60
80
100
Percentage (%)
Hyper in 4X
Hypo in 4X
Hyper in 2X
Hypo in 2X
c
Relative Methylation Level (2 Δct)
(+McrBC/-McrBC)
0.0
2.5
5.0
7.5
10.0
12.5
15.0
17.5
20.0
2x
4x
***
*
*
***
*
**
***
FT
ELF3
PRR5-2
COP1
TOC1
FKF1
SPA1
PIF3
Gene
