# Supplementary material for: Artificial autotetraploidy modulates flowering regulation in industrial hemp through DNA methylation and transcriptomic reprogramming
Source: Mol Hortic. 2026 Aug 6;6:53. doi: 10.1186/s43897-026-00245-8 (PMC13445816; doi:10.1186/s43897-026-00245-8)
Supplement: Supplementary file 5 [file 43897_2026_245_MOESM5_ESM.pdf]

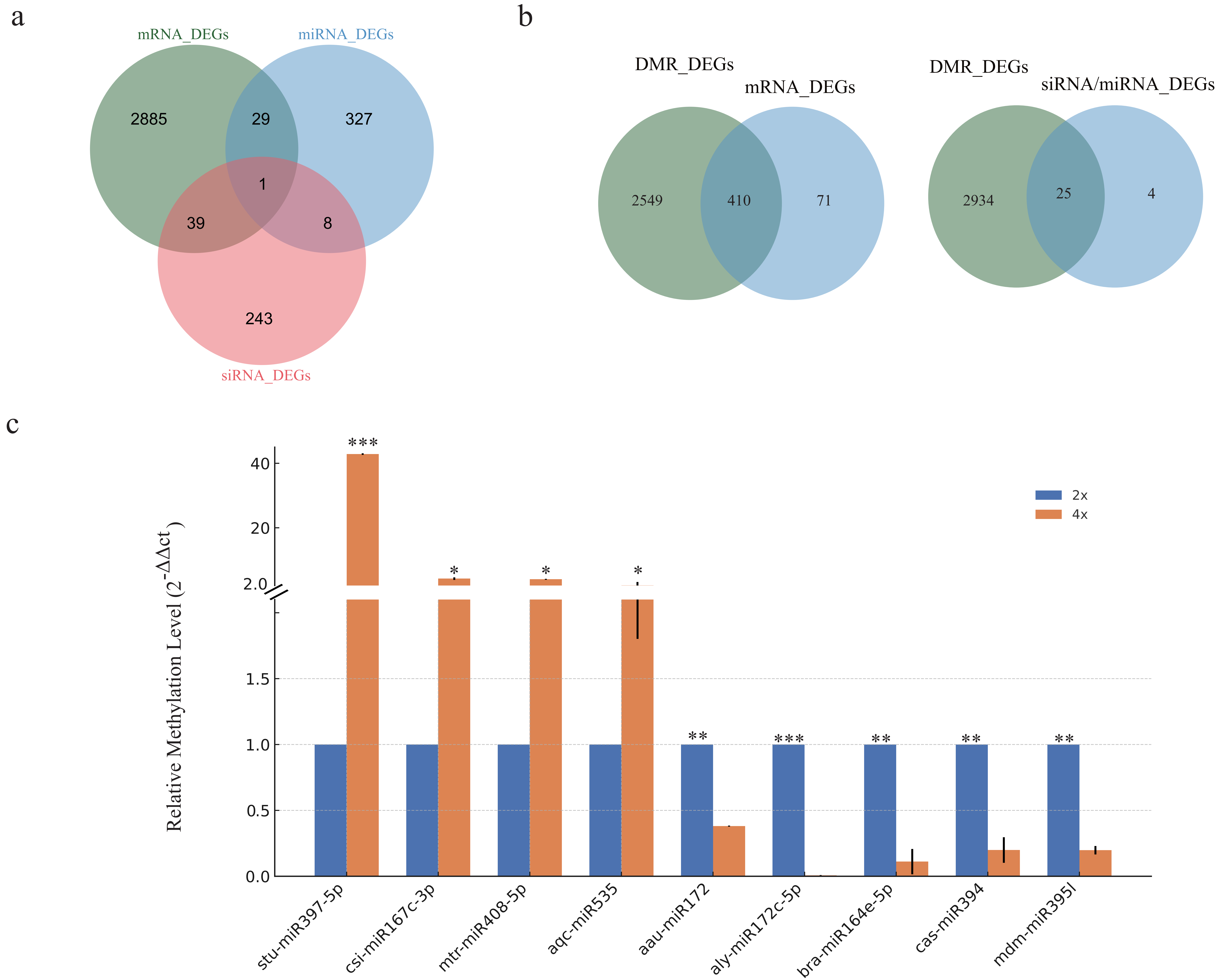
a
mRNA_DEGs
miRNA_DEGs
siRNA_DEGs
2885
29
327
1
39
8
243
b
DMR_DEGs
mRNA_DEGs
2549
410
71
DMR_DEGs
siRNA/miRNA_DEGs
2934
25
4
c
Relative Methylation Level ($2^{-\Delta\Delta ct}$)
2x
4x
***
*
*
*
**
***
**
**
**
stu-miR397-5p
csi-miR167c-3p
mtr-miR408-5p
aqc-miR535
aau-miR172
aly-miR172c-5p
bra-miR164e-5p
cas-miR394
mdm-miR395l
