# Supplementary material for: Artificial autotetraploidy modulates flowering regulation in industrial hemp through DNA methylation and transcriptomic reprogramming
Source: Mol Hortic. 2026 Aug 6;6:53. doi: 10.1186/s43897-026-00245-8 (PMC13445816; doi:10.1186/s43897-026-00245-8)
Supplement: Supplementary file 6 [file 43897_2026_245_MOESM6_ESM.pdf]

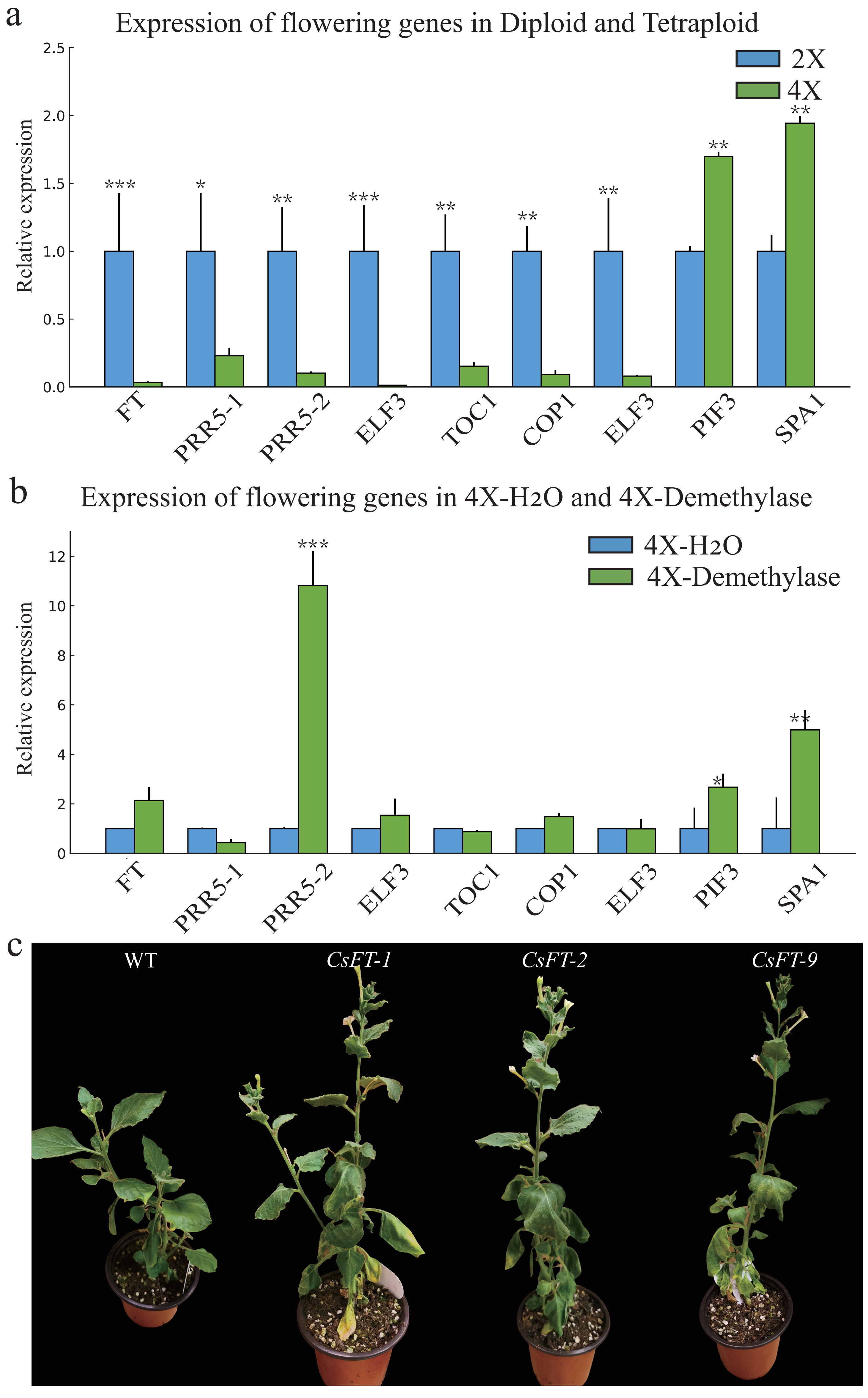
a
Expression of flowering genes in Diploid and Tetraploid
2X
4X
Relative expression
2.5
2.0
1.5
1.0
0.5
0.0
***
*
**
***
**
**
**
**
**
FT
PRR5-1
PRR5-2
ELF3
TOC1
COP1
ELF3
PIF3
SPA1
b
Expression of flowering genes in 4X-H2O and 4X-Demethylase
4X-H2O
4X-Demethylase
Relative expression
12
10
8
6
4
2
0
***
*
**
FT
PRR5-1
PRR5-2
ELF3
TOC1
COP1
ELF3
PIF3
SPA1
c
WT
CsFT-1
CsFT-2
CsFT-9
