# Supplementary material for: Artificial autotetraploidy modulates flowering regulation in industrial hemp through DNA methylation and transcriptomic reprogramming
Source: Mol Hortic. 2026 Aug 6;6:53. doi: 10.1186/s43897-026-00245-8 (PMC13445816; doi:10.1186/s43897-026-00245-8)
Supplement: Supplementary file 7 [file 43897_2026_245_MOESM16_ESM.docx]

**Table S1 Phenotypic analysis of plants of industrial hemp after maturation**

|  | Fresh weight of whole  (g) | Dry weight of  whole  (g) | Inflorescence length  (cm) | Fresh weight of inflorescence  (g) | Dry weight of inflorescence  (g) |
| --- | --- | --- | --- | --- | --- |
| Diploid | 404.42±19.03 | 87.70±10.04 | 9.37±0.82 | 3.61±0.67 | 0.77±0.20 |
| Tetraploid | 551.57±26.01 | 139.06±7.62 | 23.20±1.20 | 10.58±1.02 | 1.92±0.26 |
| Increase rate | 36%^**^ | 59%^**^ | 148%^**^ | 193^**^ | 149^**^ |

Note：** indicates a highly significant difference between diploid and tetraploid plants (*p* < 0.01).

**Table S2 Primers for RT-qPCR expression of differentials genes**

| Gene ID | Name | Sequence (5'-3') | Length |
| --- | --- | --- | --- |
| LOC115725285 | *TUB* | F:CTCGGCTGAGAAAGCATACC  R:CCATGCCTAGGGTCACACTT | 20  20 |
| LOC115696989 | *FT* | F:ATTGGGAAGGCAAACGGTGT  R:TTCCCCCAGATCCTGTCTCC | 20  20 |
| LOC115697482 | *ELF3* | F:CAGCAAGCAGTTGGAACACC  R:GCCTCATTCGTTCGGCCTAT | 20  20 |
| LOC115704169 | *PRR5-1* | F:TGAGTATCCTCCACAGCTGGA  R:CCAGGATAGAGAACGCACCA | 21  20 |
| LOC115716206 | *PRR5-2* | F:AAACCCTACATCAGCCGGTG  R:AGCTTTTTGAGGGACCAGGG | 20  20 |
| LOC115707394 | *COP1* | F:GGACATGGAAGATGCGGACA  R:GGCGTAGGTGAGTTACGAGG | 20 |
| LOC115711591 | *TOC1* | F:GTCTGCTTACAACCGGGTCA  R:TCAAAACAGCGCTCCTTCCT | 20 |
| LOC115714162 | *FKF1* | F:TCTAGAGCCCGATTTCCCCA  F:CCTTTGAGCACGAGGATCCC | 20 |
| LOC115699851 | *SPA1* | F:ATTAGGGTTGGCATCTTC  R:AAATAGGACGCACGAGGA | 18 |
| LOC115719625 | *PIF3* | F: TCTGAATGCCATAGTGAT  R:TCTCGTTAATTCTATCCCT | 18  19 |

| Name | Sequence (5'-3') | Length |
| --- | --- | --- |
| U6-F | CATCCGATAAAATTGGAACGATACAG | 26 |
| U6-R | CATTTCTCGATTTGTGCGTGT | 21 |
| mFT-F | AGACAAAATATCGGGGCGTCA | 21 |
| mFT-R | TGGATTACTCCACTCATCACCA | 22 |
| mELF3-F | GCATCGACCATGGAAACCTC | 20 |
| mELF3-R | GTGGAACCAAAGGCAAGAGT | 20 |
| mPRR5-2-F | CAAAGGGTGTTTATGAGTCCCT | 22 |
| mPRR5-2-R | CTTGCACTTTAGTACCAATCGTT | 23 |
| mCOP1-F | AAACTCGAATGAAATATCTGTGCAA | 25 |
| mCOP1-R | TTGGGTGGGAAAGCACTTAGC | 21 |
| mTOC1-F | CAACGACCTCATCCGCTCTT | 20 |
| mTOC1-R | CGACAAGAGGGTCGTTGTGA | 20 |
| mFKF1-F | GCGGTAAAACCCTTAATACTCGC | 23 |
| mFKF1-R | AGTCCAACATGGCACTTAACAC | 22 |
| mSPA1-F | GCATTTCGAAGTTCAATGTGAG | 22 |
| mSPA1-R | AGCTGAAGTGCCTAGTCCAG | 20 |
| mPIF3-F | CGAATAGGCTGCTTTGGTGC | 20 |
| mPIF3-R | TGTTAGGACCCGACCTCGTT | 20 |
| stu-miR397-5p | ATTGAGTGCAGCGTTGATGAC | 21 |
| csi-miR167c-3p | AGGTCATCTTGCAGCTTCAAT | 21 |
| mtr-miR408-5p | ACAGGGAACATGCAGAGCATG | 21 |

**Table S3 Primers for RT-qPCR assay for validating the result of whole-genome bisulfite and Small RNA-seq**

**Table S3 Primers for RT-qPCR assay for validating the result of whole-genome bisulfite and Small RNA-seq**

| Name | Sequence (5'-3') | Length |
| --- | --- | --- |
| aqc-miR535 | TGACAACGAGAGAGAGCACGCG | 22 |
| aau-miR172 | TGAGAATCTTGATGATGCTGCAT | 23 |
| aly-miR172c-5p | GGAGCATCATCAAGATTCACA | 21 |
| bra-miR164e-5p | TGGAGAAGCAGGGCACGTGCAA | 22 |
| cas-miR394 | TTTGGCATTCTGTCCACCTCC | 21 |
| mdm-miR395l | CTGAAGTGTTTGGGGGAACCC | 21 |

Figure S1. a. Flow cytometric results of diploid and autotetraploid industrial hemp. b. Comparison of chromosome numbers in the root tips of tetraploid (left) and diploid (right) industrial hemp plant. c-d. Hemp plant height and flowering patterns at different stages.

Figure S2. Autotetraploid and Diploid Industrial Hemp Flower Bud Differentiation Paraffin Sections at different stages. a-c are autotetraploid, d-f are diploid. W2-W4: apical flowering period, apical two pairs of stigma period, inflorescence flowering period. Br. Bract; Fl.b. Marginal bract; P. Perianth; C. Carpel; Flr. Floral primordium; Ov. Ovule; S. Stigma.

Figure S3. Genome-wide and gene-region DNA methylation patterns in tetraploid (4X) and diploid (2X) Cannabis sativa. a b. Methylation levels in the whole genome; e, f, g. Methylation levels Profiling for CHG, CHH and CpG sites.

Figure S4. DNA methylation variation between tetraploid (4X) and diploid (2X) hemp.

a. Distribution of differentially methylated regions (DMRs) across CG, CHG, and CHH sequence contexts. b. Ratios of hypermethylated and hypomethylated regions at CG, CHG, and CHH sites in 4X plants relative to 2X plants. c. McrBc- qPcR showing DNA methylation levels of circadian rhythm–plant pathway genes in tetraploid (4X) and diploid (2X) hemp. * *p*-value ≤ 0.05, ** *p*-value ≤ 0.01, *** *p*-value ≤0.001

Figure S5. Association of mRNA differentially expressed genes (DEGs) with differentially methylated regions (DMRs) and small RNA regulation.

a. Venn diagram showing the overlap among DEGs associated with DMRs, differentially expressed (DE) miRNAs, and DE siRNAs. b. Numbers of differentially expressed transcripts potentially regulated by DMRs and small RNAs, categorized into five major plant growth processes: auxin response, cell division, cellulose metabolic/biosynthetic processes, growth, and reproduction. c. Quantitative RT-PCR showing miRNA stu-miR397-5p, csi-miR167c-3p, mtr-miR408-5p, aqc-miR535, aau-miR172, aly-miR172c-5p, bra-miR164e-5p, cas-miR394 and mdm-miR395l expression in tetraploid (4X) and diploid (2X) hemp. The data were calculated with three biological repetitions.

Figure S6. Expression of flowering gene at tetranploid (4X) and diploid (2X) and overexpression *CsFT* promotes flowering in tobacco. a. Expression of *FT*, *PRR5*, *ELF3*, *TOC1*, *COP1*, *FKF1*, *PIF3* and *SPA1* at normal growth. b. Detection of gene expression levels after the gene demethylation treatment. c. The overexpression of *CsFT* induces early flowering in tobacco plants. * *p*-value ≤ 0.05, ** *p*-value ≤ 0.01, *** *p*-value ≤ 0.001.

**Materials and methods**

**Plant material**

The study used industrial hemp material DMG265 and its autotetraploid female line, which was developed by our research group(Tang et al., 2023). Healthy diploid and tetraploid female hemp plant tender shoots were chosen for cutting propagation, soaked in a 0.3 g/L rooting solution for 10-15 minutes, and then placed in seedling trays for cultivation. Approximately 4-5 weeks later, the plants were transplanted to pots and kept at 26±2℃ under a 16-hour light/8-hour dark photoperiod, receiving weekly fertilization during the seedling stage. After 6 weeks, plants of similar heights were transitioned to reproductive growth conditions to trigger flowering, with cultivation parameters set at 26±2℃ and a 12-hour light/12-hour dark cycle, along with weekly fertilization specific to the flowering stage.

Leaf samples were collected from the most recently fully mature nutritional leaves of the main stem, with sampling time defined between the emergence of two (W2) and three (W3) pairs of stigmas at the stem apex. Three biological replicates were obtained from the leaf tissue of the diploid hemp line DMG265 and tetraploid line 265-16 for RNA-Seq and Small RNA-Seq. Leaves of three plants of each genotype were collected and pooled to one biological replicate for DNA methylation sequencing. All sequencing work was conducted by OE Biotech Co., Ltd. (Shanghai, China).

**Ploidy identification**

Ploidy was ascertained through flow cytometry and chromosome squash methods. Leaf apices exhibiting vigorous growth were harvested and transferred to a pre-chilled (4°C) 35-mm Petri dish. After adding 1 ml of LB01 dissociation solution, the leaves were quickly fragmented with a scalpel, and the resulting liquid was filtered through a 40-μm mesh into a centrifuge tube. Subsequently, pre-chilled RNase was introduced and incubated on ice for 15-30 min. Propidium iodide (PI) staining was conducted for 15 minutes, and flow cytometric analysis was then carried out using a BD Accuri C6 instrument. Shoot tips of *Cannabis sativa* were excised and treated with a 0.3 g/L rooting solution prior to their transfer to hydroponic culture at room temperature. After about three weeks, root tips (approximately 1 cm) were rinsed and soaked in distilled water before being stored at 4°C for more than 12 hours. They were then fixed in Carnoy's solution (methanol:glacial acetic acid, 3:1) for a duration ranging from 8 to 20 hours. Fixed root tips were rinsed with distilled water, immersed in 1 mol/L HCl, and hydrolyzed at 60°C for 3-5 minutes, followed by softening in 45% acetic acid for 45 minutes. The meristematic regions of the root tips were then stained with modified carbol-fuchsin for 1 hour. Samples were gently tapped with forceps on microscope slides to evenly spread cells and determine chromosome numbers using oil-immersion optical microscopy.

**Measurement of agronomical traits**

Phenotypic measurements of hemp were carried out in accordance with the "Descriptive Norms and Data Standards for Hemp Germplasm Resources". Eight female hemp plants (diploid and tetraploid) were observed for 10 weeks. Weekly measurements included plant height, stem diameter, leaf dimensions, petiole length, leaf area, and branch number during 1-6 weeks with 16-h photoperiod. In weeks 7-10, plants were moved to a 12-h photoperiod with weekly height measurements. At flowering maturity (10% green fruit clusters or 50% wilting), inflorescence length and whole plant/inflorescence fresh weights were recorded. After drying, the total weights of the whole plant and inflorescence were measured. The timing of key flowering stages, including flowering initiation (first pistil emergence), apical flowering (presence of a single flower at the apex), inflorescence flowering (formation of three pistil pairs), and inflorescence maturation, was recorded for both ploidy levels.

**Scanning electron microscope observation of female cannabis flower structure**

Floral tissues (~5 mm) were excised from the pistil apex, fixed in 70% ethanol for a minimum of 10 minutes, dehydrated through a series of graded ethanol (90% and 100%), and subjected to critical point drying in a Quorum K850 critical point dryer using liquid CO_2_. A single stigma was affixed to a conductive stub with double-sided conductive tape and analyzed using a Hitachi SU-3500 scanning electron microscope.

**Blotting Method of Stomatal Observation**

Leaf samples from diploid and tetraploid *Cannabis sativa* were washed with distilled water, dried by blotting, and then coated with a thin, even layer of transparent nail polish, which was left to dry naturally. The nail polish film was delicately removed with fine-tipped forceps and placed on a glass slide with distilled water to prevent air bubbles. Stomatal dimensions and density were assessed by analyzing 15 random fields in each leaf sample.

**Cannabis flower bud paraffin section**

Fresh, intact female cannabis flowers at four developmental stages were promptly immersed in FAA solution at room temperature for a minimum of 24 hours. After fixation, surplus leaves were excised, and the fixative was thoroughly washed off. Samples underwent standard paraffin section preparation, involving dehydration, clearing, embedding, sectioning, drying, deparaffinization, rehydration, staining, and mounting with resin, followed by observation and photography using light microscopy.

**RNA Sequencing Data Analysis**

Total RNA extracted from Cannabis leaves was quality-checked, followed by purification, fragmentation, and reverse transcription into cDNA. After end-treatment and adapter ligation, double-stranded cDNA underwent PCR amplification to produce ample paired-end data. Sequencing was carried out on an Illumina platform following quality control procedures. Raw sequences underwent filtering to acquire clean reads, which were subsequently aligned to the Cannabis genome database (Cs10: GCA_900626175.2) using HISAT software. Differentially expressed genes were identified using the DESeq software, applying a threshold of |log₂(fold change)| > 1 and a significance level of *p* < 0.05. Subsequently, these genes were annotated and functionally classified through enrichment analyses using the Gene Ontology (GO) databases.

**Small-RNA Sequencing Data Analysis**

Small RNA libraries were constructed using a NEBNext Small RNA library prep set for Illumina kit (Cat. No. NEB#E7330S, NEB, USA) according to the manufacturer’s protocol. The small RNA libraries were finally sequenced using the Illumina Novaseq 6000 platform. By removing 5´ and 3´ adapters, the reads shorter than 15 nt or longer than 41 nt from the raw data were filtered. The sequences were aligned and subjected to the Bowtie search against Rfam v.10.1 (http://www.sanger.ac.uk/software/Rfam), rRNA, scRNA, Cis-reg, snRNA, tRNA and other RNAs were annotated and filtered. Then, cDNA sequence and Repbase database of repeat sequence were also identified with Bowtie software. The mature miRNAs were identified by aligning against miRBase v22 database (http://www.mirbase.org/), and the expression patterns in different samples were analyzed. After that, unannotated reads were analyzed by miRDeep2 to predict novel miRNAs. Based on the hairpin structure of a pre-miRNA and the miRBase database, the corresponding miRNA star sequence and miRNA mature sequence were also identified. Differentially expressed miRNAs were calculated and filtered with the threshold of *q* value < 0.05 and FC＞2 or FC＜0.5.

**DNA Methylation Sequencing Data Analysis**

DNA from diploid and tetraploid Cannabis leaves was extracted, quality-checked, and processed into sequencing fragments. After end-repair and A-tailing, sequencing adapters with complete cytosine methylation were ligated. Following bisulfite treatment and library construction, raw data were filtered using fastp to remove low-quality reads. Filtered data were aligned to the Cannabis reference genome (Cs10:GCA_900626175.2) using Bismark, with sequencing metrics including clean bases, Q30, GC content, and mapping rates calculated.

MethylKit analyzed Bismark mapping results, identifying methylation sites with ≥10× coverage and ≥1 methylated reads. Methylation levels were statistically evaluated across CG, CHG, and CHH contexts in genomic functional regions: promoters, introns, exons, and downstream regions. Differentially methylated regions (DMRs) between diploid and tetraploid Cannabis were determined based on significant methylation level differences. Differentially methylated genes were identified based on a methylation level difference of ≥10% in the regions, with a statistical threshold of *p* < 0.05. Associated genes underwent Gene Ontology (GO) enrichment analyses to elucidate functional and metabolic annotations.

**Real-time qRT-PCR**

To validate the nine significantly downregulated genes in the circadian rhythm pathway identified in the analysis, real-time quantitative PCR was conducted. *Tubulin* (*TUB*) was chosen as the internal reference gene, and primers were designed with Primer5 and synthesized by Hunan Qingke Biotechnology Company (primer sequences are provided in Table S2).

Nine miRNAs were also selected for qRT-PCR for comparison with sequencing data. The miRNAs were extracted from each sample using EASYspin Plant microRNA Kit (Aidlab Biotechnologies, Beijing, China). The miRNA First-Strand cDNA Synthesis Kit (Aidlab Biotechnologies, Beijing, China), which is based on a poly-adenylation protocol was used for mature miRNA reverse transcription, and miRNA Real-Time PCR assay kit (Aidlab Biotechnologies, Beijing, China) was used for real-time qPCR. The cDNA of the small nuclear RNA U6 was used for RNA normalization. (primer sequences are provided in Table S3).

McrBC-qPCR

The methytation level was examined by McrBC-qPCR. McrBC is an enzyme that cuts most methylated DNA. The Modified CTAB Plant DNA Kit (Aidlab Biotechnologies, Beijing, China) was employed to isolate total DNA from the leaves. McrBC digestion was performed exactly according the manual (Takara Biomedical Technology, Beijing, China). qPCR procedure was conducted as above described. The primer sequences are provided in Table S3. The relative methylation levels detected by McrBC-qPCR were calculated by comparing digested samples to their corresponding undigested controls.

**Demethylase Assay**

Six 4X individuals at the W2-W3 stage were randomly chosen for methylation validation.The youngest fully emerged leaves of three plants' main stems were treated daily for 7 days with 50 μM 5-azacytidine, a cytosine analog known to decrease DNA methylation and H3K9 dimethylation both in interphase and mitosis. The other three plants were used as controls and treated with water. Transcription levels of specific genes, including *CsFT*, were compared between the control and treatment groups using qRT-PCR.
